# Surgical Cases

**Published:** 1839-01-12

**Authors:** William Harris


### SURGICAL CASES.

Reported by William Harris, M. D.

*Case of Incised Wound of the Wrist Joint,—healed by the first intention.*

J. R., a son of a respectable farmer of Chester county, aged fourteen years, whilst assisting to chop the winter's fire wood, received, accidentally, a severe and direct blow from an axe. It passed through the wrist, in a transverse direction, between the lower end of the radius and ulna, and the upper surface of the first carpal row. The blow divided all the soft parts that covered the bones, except the radial artery, the tendons of the extensor muscles of the thumb, and of the flexor carpi radialis, with about an inch of integuments which covered them in front.

When I took hold of the arm and raised it up to examine the wound, the hand fell forward, as a loose appendage, the palm in contact with the front part of the fore-arm, which completely exposed the radio-carpal articulation, and showed that the axe had divided also a slice of cartilage and bone from the converse surface of the scaphoides, lunare, and cuneiform bones, which slice did not fall out, but remained in contact with the radius. What was to be done? The boy's family, and the bystanders, united in the opinion that it was idle to attempt to save the hand, as it was now attached only "by a piece of skin." The boy himself was passive. I promptly decided, however, as fingers and toes had united, after having been entirely severed, that there was reason to hope for success in this case, especially as we had one undivided artery, and at least an inch of integument. I accordingly proceeded to arrest the hæmorrhage; after which I adjusted the slice of cartilage, and brought the cut surfaces exactly apposite, and united them by the interrupted suture, putting four stitches upon the back part of the wrist, and two in front. After placing a compress of lint in front, and another upon the back of the wrist, I commenced with a roller at the fingers, and continued it, by circular and reversed turns, as high as the elbow; the fore-arm at the same time being flexed upon the arm; then two splints were applied, one upon the fore and the other upon the back part of the arm, and the roller brought down over them to the fingers again. This dressing remained undisturbed until the fifteenth day, the boy, in the mean time, being kept quiet, and strictly dieted. Very little pain or inflammation followed the injury. On the fifteenth day, whilst I was removing the dressings, the patient and his family were very apprehensive that we should find the hand entirely detached, but were agreeably surprised when they discovered, after all the dressings were removed, that the arm and hand were in a very healthy condition, and that the adhesions already formed between the two, were pretty firm. I now, after washing the wound clean, and carefully drying it, applied, longitudinally, a strip of adhesive plaster, a half an inch wide and a foot long, between every two stitches, after which I cut and removed them. The wound, from this time forward, was dressed every two days, with the adhesive strips, simple cerate, lint, roller and splints, until it was completely healed, which was accomplished in about six weeks. The hand for several months after was cold and useless. By frictions, however, with warm oil, and passive motions of the joints, at the end of one year he could take hold of large objects with the same hand, and at the end of two years he declared that it was as perfect as it had ever been, and that the wrist, though rather inflexible, had in it sufficient motion for all useful purposes.

*Case of lacerated wound of the ear, in which the whole cartilaginous portion was separated from the temporal bone,—healed by the first intention.*

M. S., aged four years, the daughter of a farmer, was carelessly placed, by a labouring man, upon a seat over a roller, whilst rolling the sward grounds, in the spring of the year. She fell from her seat, and the edge of the roller scraped from the temporal bone the whole of the cartilaginous portion of the ear, and the skin from about one inch above it. I found it hanging by the soft and pendulous portion, and soiled with dirt and blood. The mangled condition of the ear, under ordinary circumstances, would have induced me to remove it, but the success of the wrist case encouraged me to make another effort at restoration. I accordingly washed away the dirt, carefully restored the organ to its original situation, and secured it there by the interrupted suture. Union by the first intention took place, and the parts were firmly united in about two weeks. The meatus auditorius externus was reduced to about one-half its usual diameter, in consequence of the imperfect apposition of the parts. This might have been prevented, by the introduction of a sponge tent into the meatus during the healing process. The hearing, however, was perfect.

*Case of lacerated wound of the Ear.*

A boy, aged sixteen years, was sitting in a cart, driving a pair of oxen that were attached to it. Owing to his imperfect control of the animals, one of the wheels passed over a high stump, upset the cart, and caught his ear between the edge of the vehicle and a stone, and removed the whole of the external organ, *pinna* and *lobus*, leaving the auditory process of the temporal bone without integument. I did not attempt a restoration of the removed part, as I had no confidence at that time in such an effort. This decision, however, I have often regretted, as a reunion even in that case was possible. The lacerated surface was dressed with simple cerate, lint, a compress and roller. It was upwards of two months before the cicatrization was complete. The chief difficulties in this case arose from disposition in the granulation to close over the external meatus, as the skin which lined it had been torn away for more than a fourth of an inch within the auditory process. This I succeeded in preventing, by keeping the orifice open with a sponge tent until the external and internal skins met and united. The hearing was perfect, and, by wearing his hair over both ears, the deformity was scarcely perceptible.

*Case of extirpation of the Parotid Gland.*

Mrs. J. Eve, aged forty years, sent for me on the 6th of May, 1828, to examine a tumour near her right ear. It was about the size of an English walnut, of an irregular shape, indurated, and was situated upon the angle of the lower jaw, extending a little in front of the lobe of the ear, and dipping down deep between the ramus of the jaw and the mastoid process of the temporal bone. She consented to have the tumour extirpated, and accordingly I called upon her the next day, accompanied by two of my medical students. I commenced the operation by making a perpendicular incision from a point in front of the external meatus to the angle of the jaw. There was no difficulty in tracing the tumour, as it was of much more solid consistence than the surrounding parts. After I had dissected from the angle of the jaw that part of the tumour that lay on the front of it, I proceeded very cautiously with the dissection, at one time behind the ramus, and again in front of the mastoid process—not being interrupted by hæmorrhage from any vessel that required a ligature. At length I reached the base of the tumour, and, in making the last stroke of my knife, a considerable artery spouted forth its blood, which, I apprehend, was the external carotid. In passing a ligature around this vessel I included a small twig of a nerve, which gave her considerable pain at the time, and continued to torment her until the ligature came away. The external wound was united by the interrupted suture, and adhesive straps, and dressed in the usual way, with lint, compress and roller. Upon calling the next day, I found every thing doing well, the pain from the ligature excepted. I observed, however, as might have been expected, that a paralysis had occurred in the face, the corner of her mouth, next the side upon which the operation had been performed, being now exactly in front of her front incisor teeth, and the opposite corner near the opposite ear. The wound, however, united perfectly in about three weeks. The paralysis of the side of the face was clearly owing to my having divided the facial nerve. I encouraged her to expect that in time the present deformity would be removed, and directed her, as often as she was at leisure, to press her hand against the paralysed cheek, and at the same time to push it towards the ear, so as to bring the mouth into its natural situation. This direction was faithfully observed, and one year after the operation, I saw her with her mouth in its natural position. This tumour, after its removal, I decided to be the parotid gland. Its appearance, size, and location, the magnitude and depth of the artery divided, and the subsequent paralysis of the side of the face, all led to that conclusion. Besides, the cavity usually occupied by the parotid gland was now empty. But whether I removed every particle of that organ, or whether such an effort be practicable, it is not my purpose here to contend. It is sufficient that the operation was completely successful, the patient having since enjoyed good health, without any return of the disease.

*Case of ununited Fracture and dry Gangrene, cured by Amputation.*

A. B., of Montgomery county, aged seventy-two years, in attempting to stop a pair of horses that were running away with a wagon, fell down, and one of the wheels passed over his right leg, and broke it. Dr. J. Huddleson, of Norristown, was called to the case, and reduced and treated the fracture in the usual way. The eighth week after the accident, he invited me to a consultation in the case. Upon examination, I found that not the slightest bony union had taken place, and that *dry mortification* had commenced in his toes, and was extending up the foot. We decided that an amputation of the limb was the only means that could save his life. To this the old man gave a reluctant consent, and two days afterwards I performed the circular operation below the knee, in the presence of Doctors Huddleson, Thomas, and Smith. The operation was successful. The stump, considering the age of the patient, healed rapidly, and was not even threatened with the gangrene that had assailed the foot. In less than three months after the operation, he was travelling abroad upon crutches, in good health. This case may be added to the cases of Professor Gibson, Dr. Carmichael, and others, in favour of amputation in cases of dry gangrene.

*Case of fracture of the neck of the Thigh-bone, cured; and of fracture of the Leg, successfully treated by an immoveable apparatus.*

Mrs. C. D., of Montgomery county, aged seventy years, in climbing over a fence, fell and broke the neck of her right thigh-bone. The fracture was treated in the ordinary way, and with the ordinary want of success. The eighth week after the accident, she crawled out of bed, placed the lame limb upon a chair, whilst she sat upon another, and commenced spinning, turning her wheel with her sound leg—declaring that as she had one sound limb, and a whole heart, she could still "earn her bread by the sweat of her brow." She ordered a pair of crutches, that she might be enabled, as usual, to carry home to the neighbouring farmers the thread that she had spun, and receive her wages. She ordered, moreover, a high-heeled shoe, as the lame limb was now two inches shorter than the other. Accordingly, after a few days, with the aid of the high-heeled shoe and her crutches, she set out with a bundle of thread upon her back, in high spirits; but she had scarcely got beyond the precincts of her humble cottage, when the high heel, sinking into a hole in the ground, and sticking fast, she fell, and broke the tibia and fibula of the same limb, a short distance above the ankle. "Doctor," said she, as I entered her door, "I have been again unlucky,—I have broken the same leg above the ankle,—do what you can for me, but I cannot go to bed again,—the spinning is promised, and I must keep my word." I determined, as far as practicable, to accommodate her; and, to that end, I procured two thick binder's boards, (pasteboard,) each three inches wide, and long enough to reach from her knee to the bottom of her foot. I soaked them in boiling water until they were quite soft and pliable, then having placed my patient upon her back in bed, and the broken fragments of her limb being properly adjusted, I caused an assistant to hold it in an elevated position, while I applied a roller, commencing at her toes, and carrying it up, by circular and reversed turns, as high as the knee. I next applied the splints, moulding them carefully to the inequalities of the limbs, and brought the roller down over them to the foot again. The next day finding that the splints had become dry and firm, I allowed her to creep out of bed carefully, and to place the fractured leg upon a bench, whilst she sat upon her chair, singing her usual merry song and turning her wheel with her sound foot. She continued her daily avocation in this way for upwards of six weeks, at the end of which time, finding that bony union had taken place, and that the limb was perfectly straight, I removed the splints. She did not venture out again upon her crutches, however, until four weeks had elapsed after the removal of the splints; but at the end of this time, she again set forth, upon a fine spring day, and made her way without difficulty, except that the injured limb swung by her side as a useless appendage, no union having taken place as yet at the neck of the bone. In this way, she attended to her usual round of duties. At the end of three months, however, after her second outset upon her crutches, the neck of the bone was evidently forming a pretty firm union and at the end of one year she could walk five miles in a day without crutches, and with no other assistance than *the high heeled shoe* and her *faithful old staff*,—nor did I think she derived more support from it than before she was injured. When I last saw her, she was in high health and spirits, and full of thankfulness.

I have attended a number of fractures of the neck of the thigh-bone, all in aged females, and this is the only one in which a cure was effected. Is it not probable, however, that if some of the old ladies in our city, now, confined to their chambers with a similar injury, were to rival the energies, and imitate the exertions of the old spinster, their efforts might lead to the same happy result?

The success of this treatment of *the fracture of the leg* is calculated to strengthen our confidence in the treatment of fractures, by the "*methode par compression inamovible*," which has been in successful experiment for several years, by the French and German surgeons.*

* That the "appareil inamovible" is not original with its present claimants (Messrs. Velpeau and Seutin) will be discovered on the perusal of the following extract from a work of a celebrated English lithotomist. Speaking of that species of club-foot called varus, he says: "After this, having another case of this kind under my care, I thought of a much better bandage, which I had learnt from Mr. Cowper, a bone-setter at Leicester, who set and cured a fracture of my own cubit when I was a boy at school. His way was after putting the limb in a proper posture to wrap it up in rags dipped in the whites of eggs, and a little wheat flower mixed; this drying, grew stiff, and kept the limb in a good posture. And I think there is no way better than this in fractures, for it preserves the position of the limb without strict bandage, which is the common cause of mischief in fractures. When I used this method to the crooked foot, I wrapt up the limb almost from the knee to the toes, and caused the limb to be held in the best posture till the bandage grew stiff, and repeated the bandage once a fortnight."—*Cheselden's Anatomy*, 11*th ed.*, *Lond.* 1778, *p.* 38.—[Eds.]

